# Extended therapy breaks from VEGFR TKI therapy in renal cell carcinoma: Sometimes less is more

**DOI:** 10.18632/oncotarget.23005

**Published:** 2017-12-06

**Authors:** Haris Zahoor, Brian I. Rini, Moshe C. Ornstein

**Affiliations:** Taussig Cancer Institute, Cleveland Clinic, Cleveland, OH

**Keywords:** renal cell carcinoma, VEGFR, TKI, PD, intermittent

The treatment landscape of metastatic renal cell carcinoma (mRCC) dramatically changed with the introduction of vascular endothelial growth factor receptor (VEGFR) tyrosine kinase inhibitors (TKI) [1]. Despite major advances in the field of immunotherapy, TKIs remain a fundamental therapy in mRCC. Novel methods to optimize VEGFR TKI delivery are thus critical to improve clinical outcomes while minimizing toxicity.

A primary challenge with TKIs is the balance of clinical efficacy and associated toxicities of long-term therapy. Treatment breaks were incorporated into the development of VEGFR TKIs to limit toxicities, suggesting that continuous treatment is not always necessary and that extended periods off treatment might be feasible without compromising clinical outcomes. Retrospective and prospective data support the feasibility of intermittent TKI dosing with extended breaks.

A retrospective analysis investigated whether patients with mRCC treated with TKI (sunitinib or sorafenib) who achieve complete response (CR) on treatment, can take a treatment break until relapse [2]. Of 53 patients who stopped treatment, 29 (55%) remained without recurrent disease at a median follow up of 8.5 months. The majority of the remaining 24 patients who relapsed were able to reinitiate the same TKI and maintained antitumor response, indicating TKIs can be stopped and restarted in select patients.

Similarly, the impact of treatment breaks of 3 months or longer for reasons other than progressive disease (PD) in mRCC patients receiving VEGFR TKI was evaluated in a retrospective study of 112 patients [3]. The median duration of the initial break was 16.8 months with a range of 12.5-26.4 months off therapy. Achievement of CR prior to the initial treatment break (n=15) was associated with a longer surveillance period (p=.0004). These retrospective data further indicate that TKI treatment breaks are feasible in some patients.

The concept of prolonged treatment breaks was prospectively investigated in a randomized discontinuation trial of sorafenib, in which mRCC patients with stable disease after 12 weeks of sorafenib were randomly assigned to receive placebo or to continue sorafenib. The progression free survival (PFS) in patients who were assigned to placebo but then crossed over at progression to restart sorafenib, was similar to patients who were continued on sorafenib. Patients who were in the placebo arm had more tumor growth but the antitumor effect of sorafenib was maintained upon reinitiating treatment further highlighting that extended breaks from therapy do not necessarily compromise clinical outcomes [4].

Additional prospective data supporting the feasibility of intermittent TKI dosing with extended breaks in patients with mRCC was recently published [5]. Patients with treatment-naïve mRCC were treated with 4 cycles of sunitinib and then restaged. Patients who had >10% reduction in tumor burden (TB) were taken off therapy. These patients then underwent imaging every two cycles and resumed therapy only if there was an increase in TB >10%. Following treatment reinitiation, treatment would again be held for >10% TB reduction. This intermittent treatment schedule was continued until progressive disease (PD) or unacceptable toxicities. Of 37 patients, 20 patients had >10% TB decrease and all patients (100%) entered the intermittent phase. The median duration of the treatment breaks was 8.3 weeks (range, 4.7 to 192.1 weeks) with seven patients having prolonged treatment breaks lasting 3.2 to 43.6 months. The clinical efficacy in this intermittent treatment trial (objective response rate (ORR) of 46% and median PFS of 22.4 months) was no worse than previously reported data in first line treatment with sunitinib [6, 7], thus suggesting that intermittent treatment with sunitinib is feasible and doesn’t compromise clinical activity in carefully selected patients.

A phase II/III clinical trial in UK is ongoing with a goal of randomizing 1000 mRCC patients to standard dosing or intermittent dosing of sunitinib [8]. The overall aim of the trial is to determine whether intermittent dosing schedule is non-inferior to standard dosing in terms overall survival and quality of life. Results of this trial will further solidify the existing data to support intermittent dosing schedule of sunitinib.

In summary, TKIs have changed the treatment landscape of RCC and will continue to play an important role in the treatment of RCC in the future. Challenges in TKI delivery include mitigating long-term toxicities while maintaining clinical efficacy, and strategies like prolonged treatment breaks are critical to optimize the delivery of TKIs.
